# Glass-Ceramic Coating on Silver Electrode Surface via 3D Printing

**DOI:** 10.3390/ma16083276

**Published:** 2023-04-21

**Authors:** Lilin Yang, Dongzhi Wang, Guoxiang Zhou, Zhidan Lan, Zhihua Yang

**Affiliations:** 1 School of Materials Science and Engineering, University of Jinan, Jinan 250024, China; 2 School of Materials Science and Engineering, Harbin Institute of Technology, Harbin 150001, Chinayangzh@hit.edu.cn (Z.Y.)

**Keywords:** glass-ceramic coating, 3D printing, sintering, silver electrode, dielectric properties

## Abstract

Silver electrodes are commonly used as a conductive layer for electromagnetic devices. It has the advantages of good conductivity, easy processing, and good bonding with a ceramic matrix. However, the low melting point (961 °C) results in a decrease in electrical conductivity and migration of silver ions under an electric field when it works at high temperatures. Using a dense coating layer on the silver surface is a feasible way to effectively prevent the performance fluctuation or failure of the electrodes without sacrificing its wave-transmitting performance. Calcium-magnesium-silicon glass-ceramic (CaMgSi_2_O_6_) is a diopside material that has been widely used in electronic packaging materials. However, CaMgSi_2_O_6_ glass-ceramics (CMS) are facing tough challenges, such as high sintering temperature and insufficient density after sintering, which significantly confine its applications. In this study, CaO, MgO, B_2_O_3_, and SiO_2_ were used as raw materials to manufacture a uniform glass coating on the silver and Al_2_O_3_ ceramics surface via 3D printing technology followed by high-temperature sintering. The dielectric and thermal properties of the glass/ceramic layer prepared with various CaO-MgO-B_2_O_3_-SiO_2_ components were studied, and the protective effect of the glass-ceramic coating on the silver substrate at high temperatures were evaluated. It was found that the viscosity of the paste and the surface density of the coating increase with the increase of solid contents. The 3D-printed coating shows well-bonded interfaces between the Ag layer, the CMS coating, and the Al_2_O_3_ substrate. The diffusion depth was 2.5 μm, and no obvious pores and cracks can be detected. According to the high density and well-bonded glass coating, the silver was well protected from the corrosion environment. Increasing the sintering temperature and extending the sintering time is beneficial to form the crystallinity and the densification effect. This study provides an effective method to manufacture a corrosive-resistant coating on an electrically conductive substrate with outstanding dielectric performances.

## 1. Introduction

In recent years, using silver (Ag) as a conductive layer material has attracted widespread attention in many fields. Although Ag has excellent physical and chemical properties, such as good electrical conductivity, easy processing, and outstanding oxidation resistance, the shortcomings, including ions migration, vulcanization in air, and low operating temperature, are also distinctive when it was used in an aggressive environment [1,2,3]. Previous studies have demonstrated that surface coating is an effective method to mitigate the above-mentioned challenges of Ag electrodes. The surface coating method can form an alloy layer or infiltration layer on the metal surface to restrain the chemical reaction between the silver and the exposed environment. Although surface coating is an effective way to protect the metal substrate, it will also sacrifice the plasticity, hardness, workability, or its unique properties. Therefore, the surface coating has become a commonly used method to protect the metal substrate, especially in a high-temperature working environment.

Glass-ceramic is a composite material composed of a large number of small-sized ceramic grains uniformly distributed in the glass phase [4]. The difference between glass-ceramic material and glass is that glass is completely amorphous, while glass-ceramic material is composed of a crystal phase and a glass phase. In a ceramic material, the crystal ceramic phases are directly produced in the sintering process; however, in a glass-ceramic material, the crystal phases are produced by the precipitation of crystal grains in the glass phase by uniform nucleation or non-uniform nucleation and gradually growing process [5,6]. The observable benefits of glass-ceramic composites include good stability, adjustable thermal expansion coefficient in a wide range, good mechanical properties, good airtightness, high strength, excellent dielectric properties, chemical stability, and high-temperature thermal stability. These advantages ensure that glass-ceramic materials have been widely used in aerospace, electronic instruments, biological sciences, and machine engineering [7]. It was reported that dual phases composites can be prepared by the sintering method, melting method [8], sol-gel method [9,10], and welding or polymer adhesion [11,12,13]. So far, a few glass-ceramic systems are available to meet the needs of different application conditions [14,15,16], including Li_2_O-Al_2_O_3_-SiO_2_(LAS) [17,18,19], MgO-Al_2_O_3_-SiO_2_(MAS) [20,21], BaO-Al_2_O_3_-SiO_2_(BAS) [22,23], CaO-B_2_O_3_-SiO_2_(CBS) [24,25], and CaO-MgO-SiO_2_(CMS) systems.

The main crystal phase of diopside (CaMgSi_2_O_6_) can be obtained by using CaO, MgO, and SiO_2_ as raw materials. Diopside phase glass-ceramic has an interwoven structure, which has excellent dielectric and thermal properties, magnificent wear and corrosion resistance, and outstanding mechanical properties [26]. Due to the facile manufacturing process and low cost of the raw materials [27], the CMS system has been widely used in many areas, such as sealing materials for solid oxide fuel cells [28,29] and bioactive materials [30].

The complex anion of the CaMg[Si_2_O_6_] diopside is the single chain of [Si_2_O_6_]^4+^. The long chain extends infinitely along the c-axis by the connection of Ca^2+^ and Mg^2+^. In this chain structure, the ions on the bottom of the [SiO_4_] tetrahedron are connected by Ca^2+^, and the vertices of the [SiO_4_] tetrahedron are connected by Mg^2+^. Diopside belongs to the monoclinic crystal system, and the unit cell parameters are a = 0.971 nm, b = 0.889 nm, c = 0.524 nm, β = 105.79°. To make CMS-based glass-ceramics meet the performance requirements of wave-transparent coatings, it is critical to ensure enough diopside crystals in the composites, otherwise, the designed properties cannot be satisfied. 

The factors affecting the properties of CMS glass-ceramics can be divided into three categories: the contents of basic components [31], the addition of crystal nucleating agents, and the heat treatment system. The effect of the SiO_2_/CaO molar ratio on the structure and thermal behavior of the CaO-MgO-SiO_2_-P_2_O_5_-Na_2_O-CaF_2_ system glass components has been systematically investigated [32]. It was claimed that the crystal nucleating agent can produce phase separation in the glass structure and thus induce crystallization and produce non-uniform nucleation. The types of crystal nucleating agents include oxides [33,34,35], metals [36], fluorides [37], and a few composites [38,39]. In addition, an appropriate heat treatment method can ensure the formation of crystal nuclei, which is critical to the uniform growth of crystal nuclei in a cost-effective and environmentally friendly way [40,41]. By using a two-step heat treatment process and ZrO_2_ as a nucleating agent, the densification, microstructure, and dielectric properties of CMS glass-ceramics show good properties for LTCC microwave dielectric components and packaging applications [42]. In the coating area, glass coating that can be 3D printed was quite necessary for the expensive metal layer to protect it from corrosion and oxidation so that the service stability and lifetimes of the ceramic-based functional devices can be improved. However, the printable, highly dense, and well-bonded coating was difficult to prepare. 

In this paper, a silver layer on an Al_2_O_3_ substrate was used as the base material, CaO-MgO-SiO_2_ was used as the raw material of the coating layer, and the glass coating was presented to manufacture a uniform glass coating on the silver by 3D printing. According to the high density and well-bonded glass coating, the silver was well protected from the corrosion environment. This printable glass coating and its sintering method can be applied to ceramic-based devices to protect them from corrosion and oxidation. This work promises an effective and easy preparation of a high-quality metal protective coating layer. 

## 2. Materials and Methods

Calcium carbonate and magnesium oxide were analytically pure and obtained from Shanghai Macklin Biochemical Technology Co., Ltd. (Shanghai, China). Boric acid, terpineol, ethylcellulose, triton, castor oil, and silica were analytically pure and provided by Sinopharm Chemical Reagent Co., Ltd. (Shanghai, China). The dispersant BYK-110 was analytically pure and provided by Dongguan Heli Chemical Trading Co., Ltd. (Dongguan, China). The ceramic substrate material was Al_2_O_3_ and its main performance parameters are shown in Table 1.

Despite crystal nucleating agents being beneficial to promote the crystallization process, they also harm the dielectric properties of glass-ceramics. As a result, in this materials system, the nucleating agents were not included, and the relative content of SiO_2_ and the ratio between the CaO and MgO were constants only by using B_2_O_3_ as the balance. The main mix proportion is shown in Table 2.

The 3D printing was carried out by a modified commercial 3D printer (based on FDM 3D printer) by direct ink writing (DIW). The ink was loaded into 30 cc syringes and compacted by vibration to remove the air inside. A Fluid dispenser provided the appropriate pressure to drive and control the ink through a 0.41-mm-diameter nozzle with a speed of 10 mm/s.

**Figure 1:**
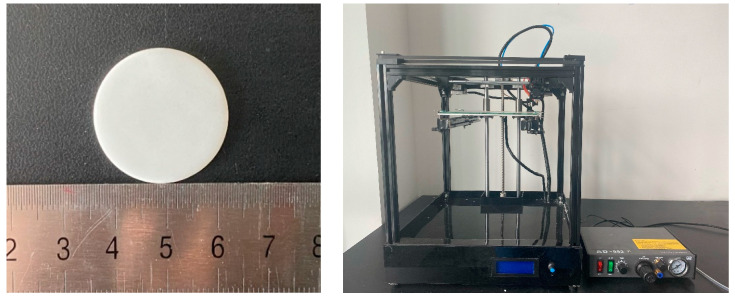
Macroscopic morphology of CMS glass-ceramic block and the 3D printer.

To prepare the bulk samples, the raw materials were ball milled by using absolute ethanol as the mixing agent. The mass ratio of the ball to powders is 6:1. The mixture was ball milled for 12 h at speed of 400 rpm, followed by drying in an oven at 80 °C for 24 h to remove the ethanol. After drying, the mixed powders were sintered at 1400 °C with a heating rate of 5 °C/min for 1 h followed by water quenching to obtain the glass-ceramic composites. After water quenching, the glass-ceramic composites were hand grounded and sieved through 75 mm. Subsequently, 4 wt.% PVA were hand mixed with the grounded powders and pressed in a steel mold with a diameter of 30 mm. The round green body was placed into a muffle furnace for sintering according to the designed sintering process. The external appearance is shown in Figure 1.

To prepare printable glass materials, an organic agent needs to be mixed with the glass powder to make a coating paste. The compositions of the organic material were designed by using terpineol as the solvent, ethyl cellulose as the binder, triton as the surfactant, and castor oil as the thixotropic agent. To obtain this organic printing agent, the binder and surfactant were mixed with the solvent terpineol, followed by heating in a water bath at 80 °C until completely dissolved. Subsequently, the diluent and thixotropic agents were added into the uniform solution, heated, and stirred until a uniform and clear solution was obtained. After mixing the organic agent and the glass powders, the mixture was heated in a water bath at 90 °C for 1 h to obtain the printable paste. 

Due the existence of the organic agent during the preparation of the coating paste, the carbon in the organic matter will produce pores during the sintering process, and thus introduce defects and break the integrity of the matrix structure, which leads to negative effects on density and physical properties of the final coating. Therefore, the coating was heated in an oven at 200 °C for 2 h followed by sintering at 500 °C for 2 h with a heating rate of 0.5 °C/min to remove the organic component. After this process, the coatings were sintered at 750 °C, 800 °C, 850 °C, 900 °C, 950 °C, and 1000 °C, respectively, with sintering times of 1 h, 2 h, 3 h, and 4 h. 

**Figure 2:**
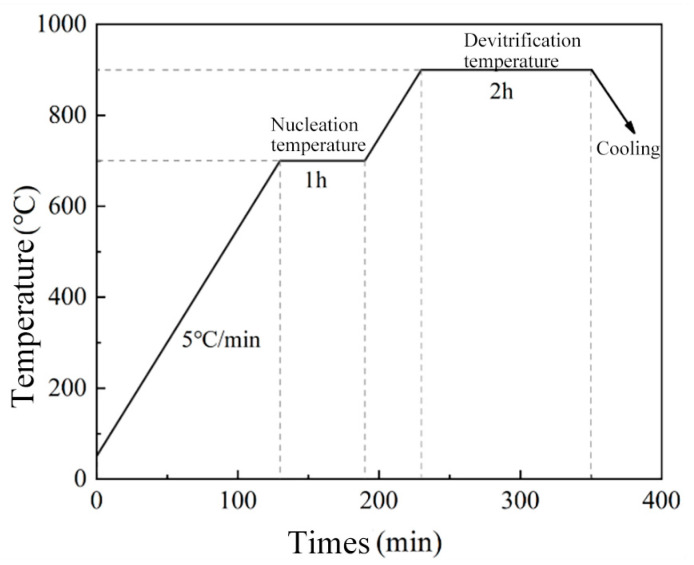
Glass-ceramic sintering process curve.

According to the nucleation temperature and crystallization temperature of the glass powder, a two-step heat treatment process was established, holding at a nucleation temperature of 700 °C for 1 h, holding at a crystallization temperature of 900 °C for 2 h with a heating rate of 5 °C/min, as shown in Figure 2. The glass powder of each component was prepared according to the sintering process to produce the corresponding CaO-MgO-B_2_O_3_-SiO_2_ glass-ceramics.

The X-ray diffraction was obtained from the Panalytical Analytical Instrument Company of the Netherlands (Almelo). The density was determined by drainage method using a high-precision balance, and the hardness was measured on the hardness tester HV-50 (Yunke, Shanghai, China) in this paper. The target material was Cu-Ka ray (λ = 0.15406 nm), with an accelerating voltage of 40 kV and loading current of 60 mA. The 2θ scanning angle was from 10° to 90°, and the scanning speed was 8°/min. The surface morphologies were observed by Zeiss Merlin Compact scanning equipment (Jena, Germany). The METTLER Thermogravimetric Analysis-Differential Scanning Calorimetry (TGA/DSC) 3+ was used to perform TG-DSC analysis, and the inert gas was pure Ar with a heating rate of 10 °C/min. The thermal expansion coefficients were tested by the German Netzsch thermal dilatometer (DIL 402C, Singapore). American Keysight E5071 network vector analyzer was used to test the dielectric properties. The German rheometer was used to test the rheological properties of the coating paste system.

## 3. Results and Discussion

### 3.1. Characterization of the Raw Materials

**Figure 3:**
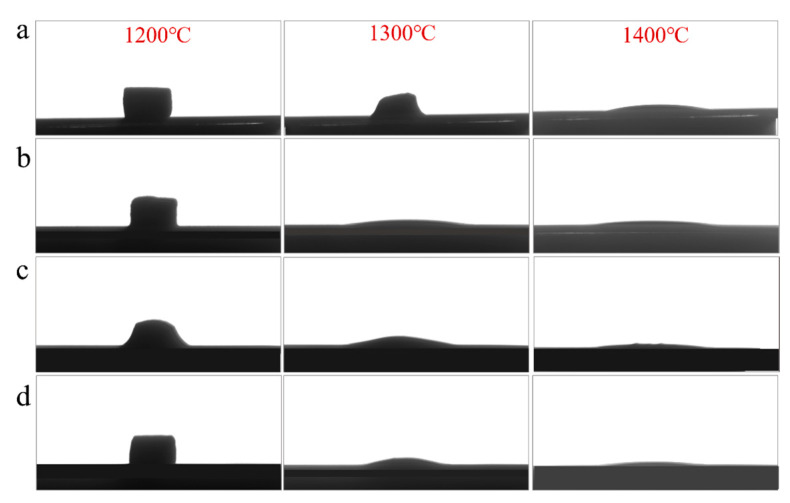
Deformation of different raw material heated at 1200 °C, 1300 °C, and 1400 °C of samples (**a**) C1; (**b**) C2; (**c**) C3; (**d**) C4.

The bulk samples were placed on an alumina substrate and sintered at various temperatures to observe the morphological change of the samples with different compositions and optimize the sintering parameters. The shape changes at different temperatures are shown in Figure 3.

It can be seen from this figure that when the sintering temperature was 1200 °C, the samples remain and maintain the original state. When the sintering temperature reaches 1300 °C, a partial melting phenomenon can be observed, and when the temperature reached 1400 °C, all samples have turned into molten liquid states. At this temperature, it can be deduced that the samples are suitable for water quenching to obtain a glassy state. Therefore, 1400 °C was selected as the sintering temperature of the raw material powder.

**Figure 4:**
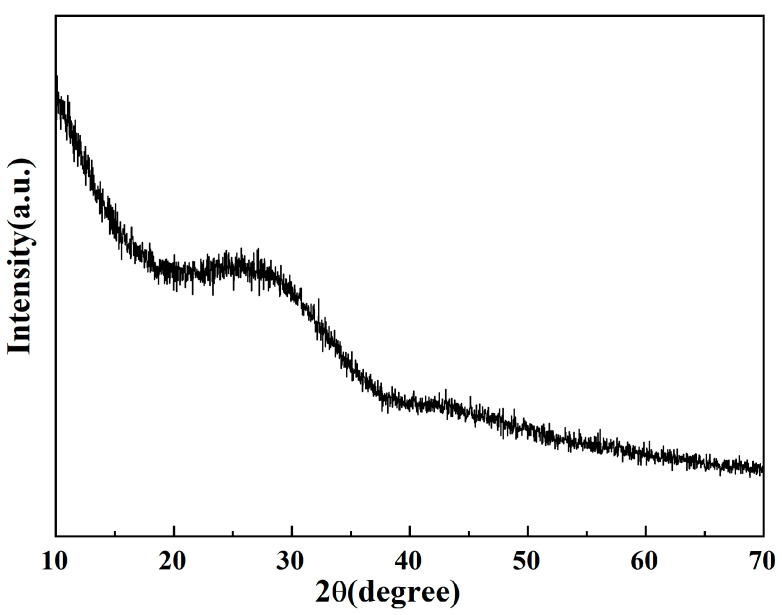
XRD diffraction pattern of glass powder.

The XRD results of the glass powder after water quenching are shown in Figure 4. It can be seen from the figure that there is a “pump peak” between 20°~37°, and no distinctive crystallization peaks can be observed in the whole spectrum, which demonstrates the amorphous state of the powders. 

**Figure 5:**
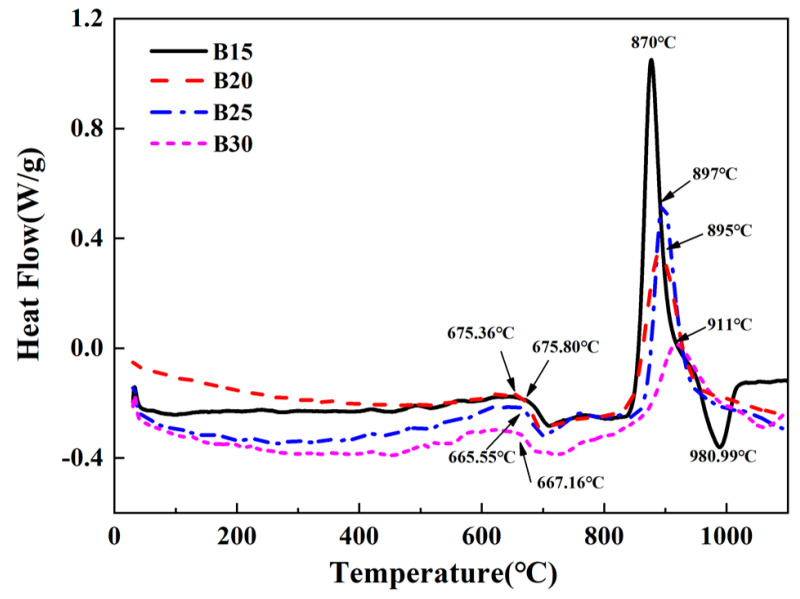
DSC curves of each glass powder component.

Figure 5 gives the DSC pattern of the glass powder with a heating rate of 10 °C/min. In the DSC diagram, downward peaks represent endothermic processes and upward peaks represent exothermic processes. It can be seen from the DSC pattern that when the temperature is lower than 600 °C, the curve is flat and no endothermic peak can be observed, indicating the thermal stability of the material in this temperature range. When the temperature was higher than 600 °C, two endothermic peaks can be observed in the C1 sample, indicating the existence of two or more crystal phases at this temperature. Except for the C1 sample, the other DSC curves have only one endothermic peak and exothermic peak, and the glass transition temperature appears at about 670 °C, and the general nucleation temperature is about 20–70 °C higher than the glass transition temperature. An obvious crystallization peak can be detected at around 900 °C, and the area of this exothermic peak is relatively large, indicating that the sample is prone to be crystallized, and the crystallization rate is the largest when it reaches the peak. It can be claimed that the increasing B_2_O_3_ content results in a decrease in the crystallization degree of the glass-ceramic materials.

### 3.2. Properties of CMS-Based Glass-Ceramics

The coefficient of thermal expansion is an important parameter to determine whether the coating and the substrate can be well bonded. If the difference in the thermal expansion coefficients between the substrate and the coating materials is distinctively large, it will cause a thermal mismatch with increasing temperatures, and result in microcracks. Generally, the difference in the thermal expansion coefficient of the coating and the substrate should not exceed 5%. 

**Figure 6:**
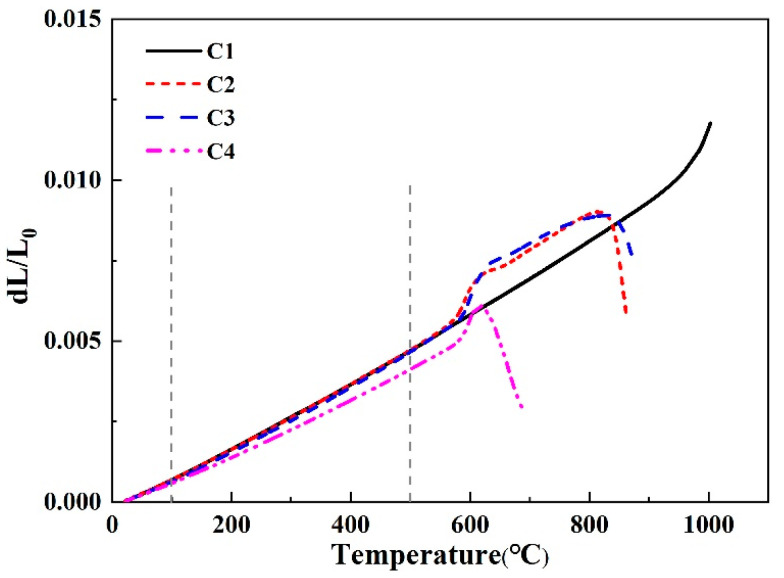
Thermal expansion curve of CMS glass-ceramics.

**Figure 7:**
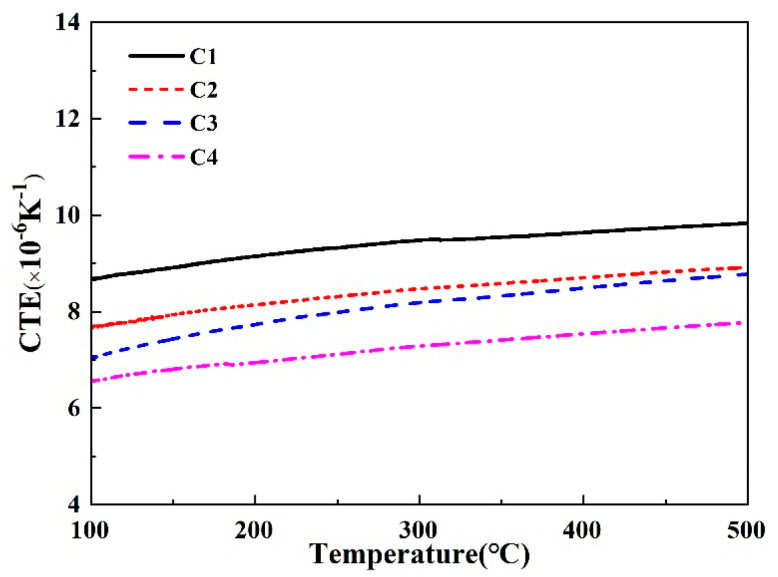
Thermal expansion coefficient of CMS-based glass-ceramics.

Figure 6 and Figure 7 present the thermal expansion performance of the CMS glass-ceramics composites. It can be seen from Figure 6, the softening point of the C1 sample reaches above 1000 °C, which is higher than that of the others. This temperature is quite high as a coating material. The softening points of other samples, however, are relatively low. The C2 and C3 are almost identical, indicating that the introduction of B_2_O_3_ will promote the formation of the liquid phase and reduce the glass transition temperature of the glass-ceramics. It can be observed from Figure 7 that the thermal expansion coefficients of all samples are in the range of 7.0 to 9.5. Among them, the thermal expansion coefficient of the C1 is the largest, and they gradually decrease with the increase of B_2_O_3_ content. The thermal expansion coefficient of the Al_2_O_3_ ceramic plate used in this experiment is 8.0 × 10^−6^ K^−1^, which is relatively close to the C2 sample.

The coefficients of the thermal expansion coefficient, Vickers hardness, and bulk density of the samples are listed in Table 3. It is also very important to have good density and mechanical properties when selecting coating materials. The nature of the crystal phase in the glass-ceramic has a great effect on the mechanical properties of the material. Even if only a small amount of crystal phase exists in the glass phase, it can sharply improve the hardness of the sample. The hardness of glass-ceramics is not only related to the type of crystal phase but also related to the size and amount of the crystal grains. Generally, the hardness of the material is proportional to the amount and inversely proportional to the size of the crystal grains. It can be seen from the table that the C1 sample has a higher bulk density and hardness, but it is not suitable as a glass-ceramic coating due to the high softening point and a mismatch in the thermal expansion coefficient. With the increase of B_2_O_3_ content and decrease of Ca and Mg contents, the bulk density of the sample gradually decreases due to the phase separation phenomenon during the sintering process, especially with boron volatilization due to the high B_2_O_3_ content. 

**Figure 8:**
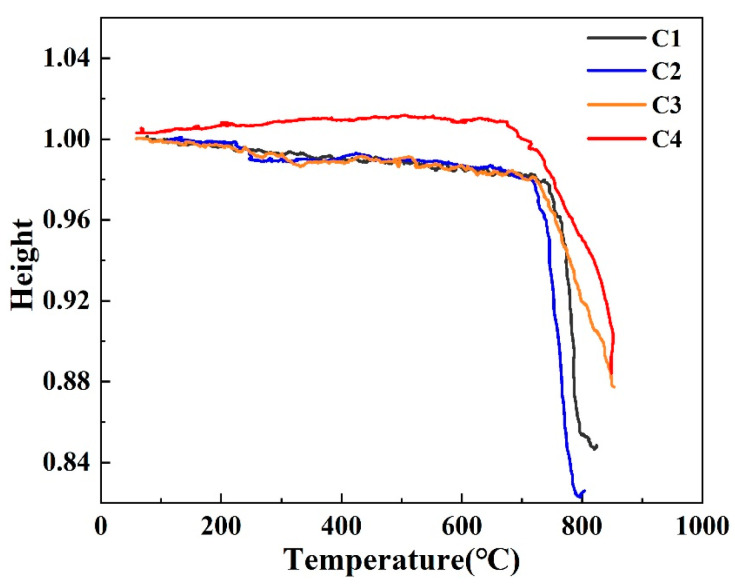
Height change of the bulk samples as a function of sintering temperatures.

Figure 8 gives the height change of the bulk samples as a function of sintering temperatures. It can be seen from this figure that the height of the samples was barely changed with the temperature lower than 650 °C. As the temperature increased, the samples gradually melted, resulting in a height reduction. Compared with other samples, the curve of the C4 sample increases first and then decreases, indicating that the sample first expands and then shrinks. This is prone to generate thermal stress in the process of sintering and generating micro-cracks. The softening point temperature gradually decreases with the increase of B_2_O_3_ content, indicating that B_2_O_3_ helps to reduce the sintering temperature of glass-ceramics.

**Figure 9:**
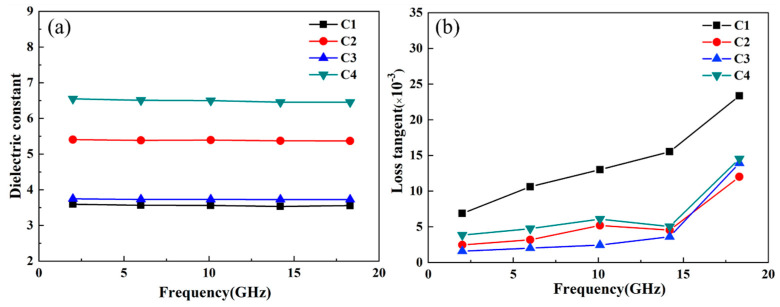
CMS dielectric properties of glass-ceramics (**a**) dielectric constant, and (**b**) dielectric loss.

Figure 9 shows the dielectric constant and dielectric loss values of the CMS glass-ceramic materials. As can be seen from Figure 9a, in the range of 0–20 GHz, the dielectric constants of C1 and C3 are relatively low. This is because the dielectric constant of the sample is often affected by its composition and structure. When the composition is constant, the dielectric properties are mainly determined by the impurities, pores, crystal grains, and lattice defects in the structure. The number of pores is governed by the other three factors, which have a great influence on the dielectric properties. In general, the higher the porosity, the lower the dielectric constant of the sample and the larger the loss tangent. It can be seen from Figure 9b that the dielectric loss of each component increases with frequency, and the dielectric loss at high frequency is higher than that at low frequency, the C3 sample has the best dielectric properties in general.

### 3.3. Performance and Microstructure Analysis of CMS-Based Glass-Ceramic Coating

#### 3.3.1. Effect of Solid Contents on Viscosity and Surface Morphology of the Coating

**Figure 10:**
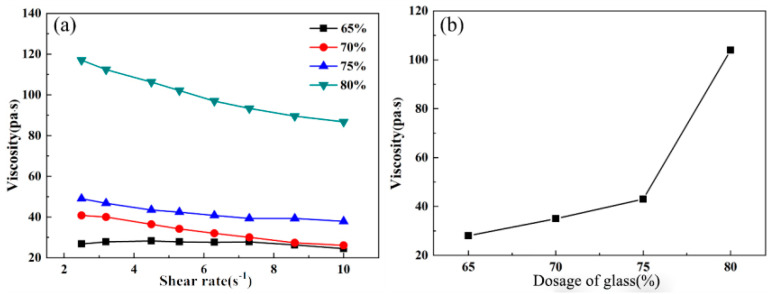
Viscosity of paste as a function of (**a**) shear rate and (**b**) dosage of glass.

Figure 10 gives the viscosity as a function of the shear rate of sample C3 (CaO = 24.5 wt.%, MgO = 10.5 wt.%, SiO_2_ = 40 wt.%, B_2_O_3_ = 25 wt.%) with various glass contents. It can be seen from the figure that the viscosities of all the pastes exhibit distinctive thixotropic behavior. Under a low shear rate, the interactions between the particles in the paste result in the shear resistance, while with an increase in shear rate, the interactions between the particles have been destroyed, and thus the particles start to rearrange their positions, which leads to the change of the viscosity of the pastes. 

**Figure 11:**
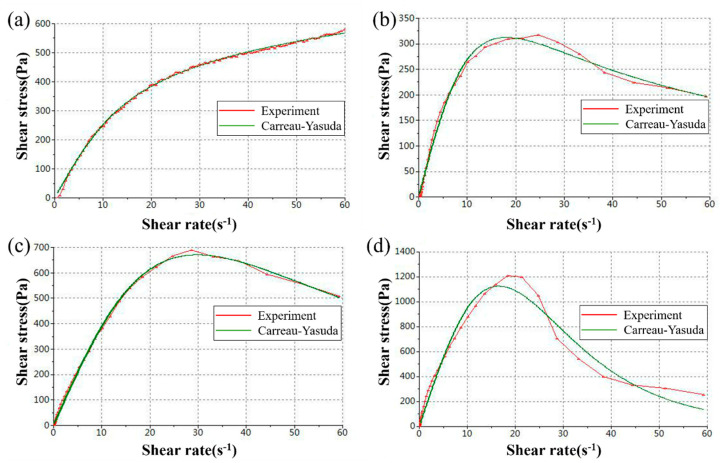
Comparison results between experimental data and theoretical model. (**a**) 65%; (**b**) 70%; (**c**) 75%; (**d**) 80%.

Thixotropic behavior is a distinct feature of non-Newtonian fluids. By comparing the experimental data with the classical fluid model, it can be found that the experimental results are consistent with the Carreau–Yasuda fluid model, as shown in Figure 11. Carreau proposed a fluid equation to describe the fluid universal curve [43]:

(1) $$\frac{{\eta -\eta }_{\infty }}{\eta_{0}{-\eta }_{\infty }}=\frac{1}{{\left[1+{\left(\lambda \gamma \right)}^{2}\right]}^{\frac{1-n}{2}}}$$

in which *η_∞_* represents the viscosity of the fluid when the shear rate is infinite, *η*_0_ represents the viscosity of the fluid when the shear rate is 0, *λ* and *n* are constants, and *n* < 1. This equation describes the relationship between the viscosity and the shear rate. In this model, when the shear rate is low, which means *λγ* << 1 and *η* = *η_0_*. While when the shear rate is high, which means *λγ* >> 1 and *η* = *η_∞_*. Based on this model, Yasuda revised the rheological formula to reflect the transition process of the universal curve. This equation is the famous Carreau–Yasuda fluid model [44]:

(2) $$\frac{{\eta -\eta }_{\infty }}{\eta_{0}{-\eta }_{\infty }}=\frac{1}{{\left[1+{\left|\lambda \gamma \right|}^{a}\right]}^{\frac{1-n}{a}}}$$

According to the curve fitting results, the results of static viscosity *η_0_* with glass contents of 65%, 70%, 75%, and 80% are 29.6 Pa·s, 36.1 Pa·s, 42.6 Pa·s, and 115.9 Pa·s, respectively.

**Figure 12:**
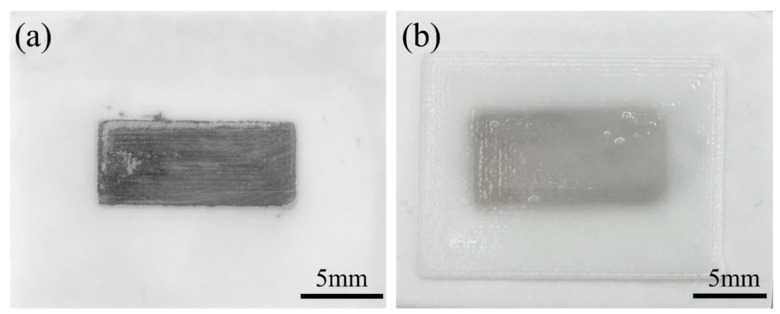
External morphologies of the samples (**a**) before and (**b**) after printing.

During the 3D printing process, the air was used as the driving force to push the paste out of the needle by squeezing the piston in the syringe, and the computer controls the printer to move according to the set path until the printing was completed. The quality of the obtained coating is not only related to the viscosity of the paste but also directly related to parameters such as applied pressure, needle diameter, and printing speed. In this study, the printing parameters remained constants, and the pastes with different solid contents were printed. Figure 12 is the external morphologies of the samples before and after printing. 

#### 3.3.2. Effect of Different Solid Contents on the Surface Morphology of the Coating

**Figure 13:**
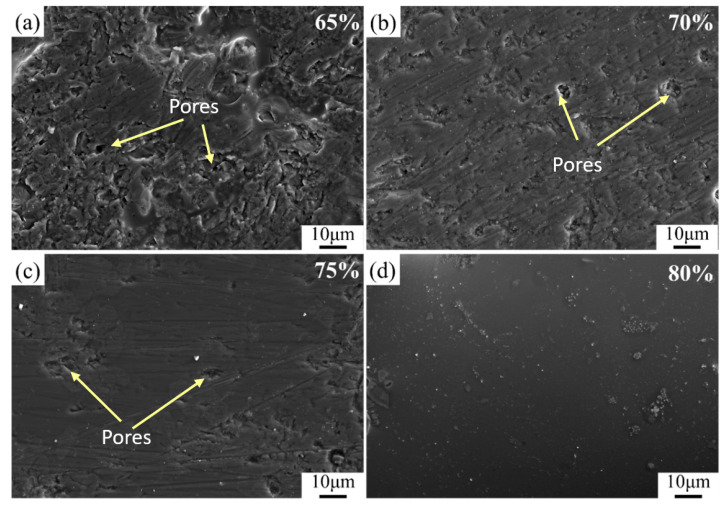
SEM images of glass ceramics with different solid content (**a**) 65%; (**b**) 70%; (**c**) 75%; (**d**) 80%.

Figure 13 depicts the SEM morphology of coatings with different solid content and sintered at 850 °C for 2 h. It can be seen from Figure 13a that when the solid phase content is 65%, a small amount of randomly distributed pores on the surface of the coating can be observed as marked in Figure 13, and the dimensions of the pores are about 1–8 μm and the solid particles are about 1–2 μm in size according to the SEM images. This originated from the volatilization of the organic components in the paste during the heating process. With increasing solid content, the surface of the coating gradually becomes smoother, and the number of pores decreases significantly. When the solid content reaches 80%, the surface has become dense without distinctive pores, as shown in Figure 13d. 

### 3.4. Interfacial Compatibility of Glass-Ceramic Coating and Ag Layer

**Figure 14:**
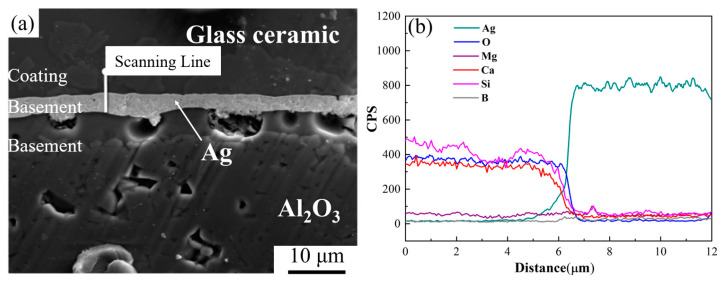
Cross-section microstructure and elements distribution of two interfaces. (**a**) microstructure; (**b**) scanning line data.

The bonding between the CMS glass-ceramic coating and the substrate is determined by various factors, including the elements’ diffusion, physical effects of hydrogen bonds and van der Waals forces, surface roughness, or substrate shrinkage. The hydrogen bonds are the best chemical bonding which has the best interface bonding force. Further, the bond force was weak by the surface roughness and substrate shrinkage which are the mechanical bonding. The van der Waals forces was the ubiquitous bonding between the coating and the substrate. The bonding by the elements’ diffusion is the best choice to improve the bonding force for the interface without any chemical bonding [45].The existence of cracks or pores will directly affect the density of the coating and cause the reduction of the performance of the silver layer. In addition, if the diffusion phenomenon occurred during the sintering process, pores will be formed at the interface between the silver electrode and the coating, or even all the silver electrodes will disappear and only pores are left, which will severely affect the performance of the electrode. To observe the bonding states of Al_2_O_3_, Ag, and glass-ceramic, the cross-section of the sample was observed and analyzed by line scanning. It can be seen from Figure 14 that there are no obvious pores or cracks at the interface between Ag and CMS coating, indicating that the interface is well bonded. Meanwhile, it can be seen from the interface energy spectrum that the diffusion depth of silver ions in the CMS glass-ceramic coating is about 2.5 μm, and the diffusion thickness is negligible.

### 3.5. Effect of Sintering Temperature on Crystallization Behavior and Microstructure of Coatings

The sintering temperature has a great influence on the precipitation and growth of crystals in the glass phase, so it is very important to choose an appropriate sintering temperature. In this section, the sintering temperature of the glass-ceramic coating was optimized, and the paste with a solid phase content of 75% was selected to prepare the coating with a sintering time of 2 h.

#### 3.5.1. Effect of Sintering Temperature on Devitrification Behavior

**Figure 15:**
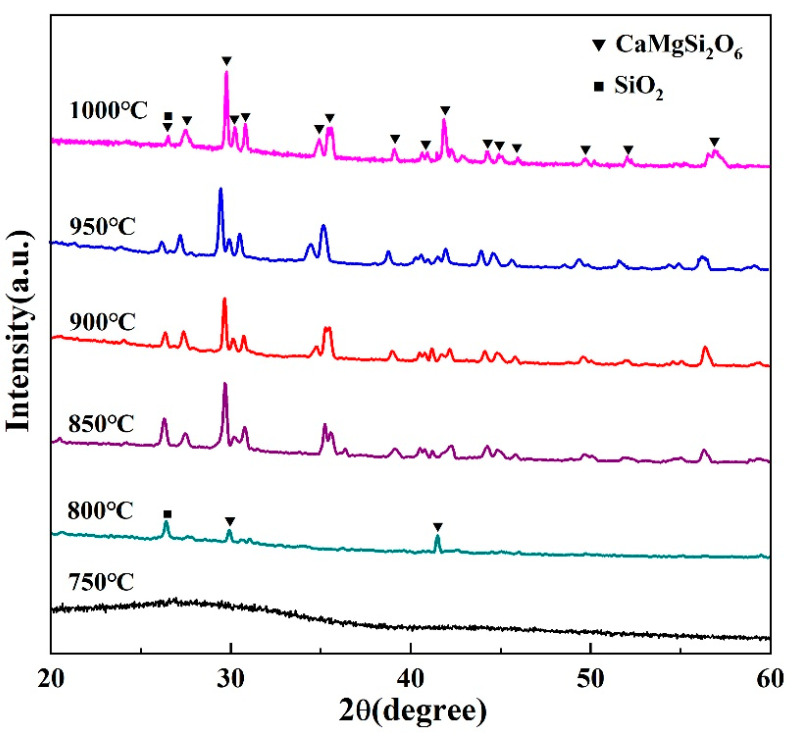
XRD patterns of CMS-based glass-ceramic coatings at different sintering temperatures.

According to the melting point and the crystallization temperature of the CMS, 750 °C, 800 °C, 850 °C, 900 °C, 950 °C and 1000 °C were selected as the sintering temperature. It can be seen from Figure 15 that when the sintering temperature was 750 °C, no diffraction peak in the XRD pattern can be detected. With the temperature increased to 800 °C, detective diffraction peaks can be observed, indicating the crystallization process at this temperature. When the sintering temperature was raised to 850 °C, considerable crystals were formed, and the diopside crystal phase existed with the α-quartz crystal phase and the glass remained the same. When the sintering temperature reached 950 °C, the diffraction peak intensity of α-SiO_2_ gradually diminished, indicating the disappearance of the α-quartz crystal, and the main crystal phase is the diopside phase. With increasing sintering temperature, the viscosity of the glass phase of the glass-ceramic decreases, and it is prone to wet the powder particles, which further increases the crystallization reaction rate and accelerates the precipitation of CaMgSi_2_O_6_ crystals. In addition, when the sintering temperature reaches the crystallization temperature, the diopside crystals precipitated and the α-quartz phase was suppressed. 

#### 3.5.2. Effect of Sintering Temperature on the Microstructure

During the sintering process, the glass powder went through a complex process, such as phase separation, nucleation, and crystallization after the temperature reaches the glass transition temperature. The sintering process was also accompanied by microstructural changes, such as particle rearrangement and pore filling. Therefore, the sintering temperature not only affects the phase composition of the coating but also has an important influence on the microstructure change of the coating. 

**Figure 16:**
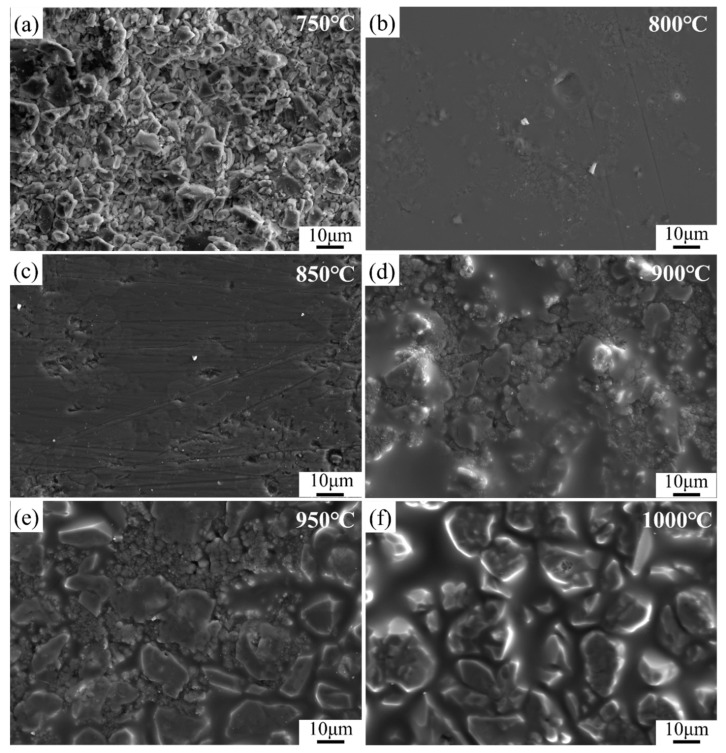
SEM images of glass-ceramic coatings at different sintering temperatures (**a**) 750 °C; (**b**) 800 °C; (**c**) 850 °C; (**d**) 900 °C; (**e**) 950 °C; (**f**) 1000 °C.

It can be seen from Figure 16 that when the sintering temperature was 750 °C, the morphology of the surface is granular and no crystal precipitation and liquid phase can be observed, indicating that the sintering temperature is too low to form a dense coating. With the sintering temperature increased to 800 °C, a liquid glass phase appears, and the surface becomes relatively dense. When the sintering temperature reaches 900 °C, massive grains can be observed in the sample and a few pores between the crystal grains and the surrounding glass phase can be observed as well. At this temperature, the viscosity of the liquid glassy phase is high, so the pores cannot be filled. With the sintering temperature increased to 1000 °C, the content of the glassy phase and the grain size gradually increase along with the disappearance of pores. However, it can be seen from Figure 16f that the silver layer has formed into silver balls after sintering, indicating that the temperature of 1000 °C is too high for the silver electrode.

### 3.6. Effect of Sintering Time on Crystallization Behavior and Microscopic Morphology of the Coating

In addition to the influence of the sintering temperature on the phase composition and microscopic morphology, the sintering time also has an important influence on the phase and morphology of the coating. In this section, the sintering temperature was fixed at 850 °C, and the effects of different sintering times on the crystallization and surface morphology of the glass-ceramic coating were investigated.

#### 3.6.1. Effect of Sintering Time on Crystallization Behavior

**Figure 17:**
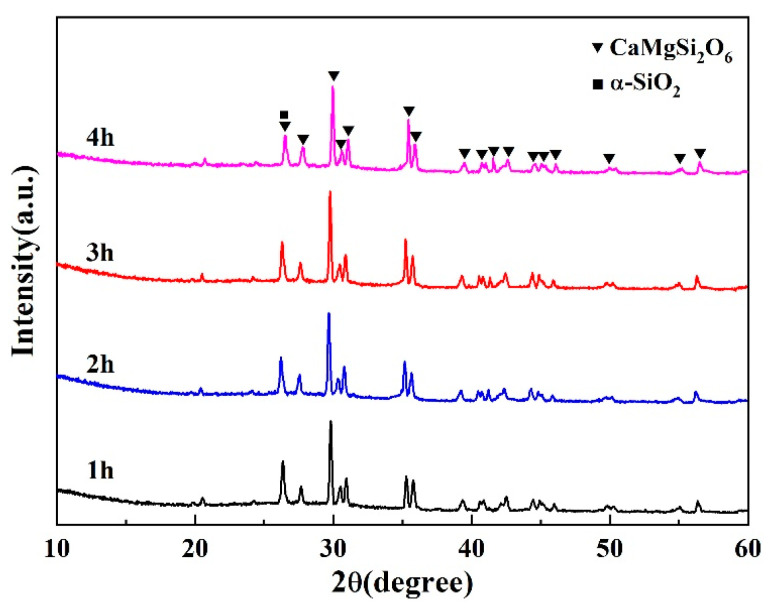
XRD pattern of glass-ceramic coating at different holding times.

Figure 17 gives the XRD pattern of the glass-ceramic coating with various sintering times at 850 °C. It can be seen from this figure that the main phases are the diopside crystal phase along with a small amount of the α-SiO_2_ phase, indicating the formation of an ideal glass-ceramic coating material. With the extension of the sintering time, the glassy phase in the coating material gradually decreased. This is due to the extension of the sintering time promoting the migration of ions, resulting in the precipitation and growth of diopside crystals and inhibiting the growth of the α-SiO_2_ phase. However, further extension of the sintering time will lead to the reduction of the glassy phase and increase the viscosity of the coating. This will lead to the reduction of ion mobility and confines the precipitation of diopside crystals. 

#### 3.6.2. Effect of Sintering Time on the Microstructure

**Figure 18:**
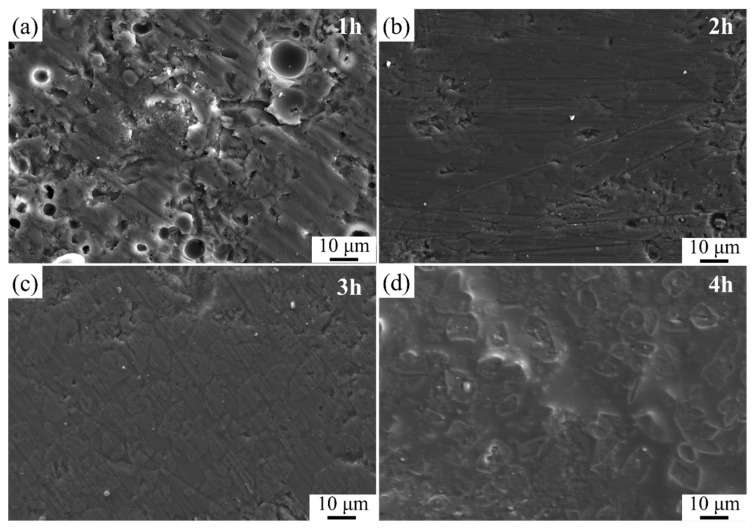
SEM images of glass-ceramic coatings at 850 °C with different holding times (**a**) 1 h; (**b**) 2 h; (**c**) 3 h; (**d**) 4 h.

Figure 18 depicts the SEM images of glass-ceramic coatings sintered at 850 °C with a sintering time of 1 h, 2 h, 3 h, and 4 h. It can be seen from this figure when the sintering time was 1 h, the densification process of the coating was not completed. With the extension of the sintering time to 2 h, it can be observed that the internal pores were gradually filled by the crystal grains. Further extending the sintering time, the size and number of the crystal grains increase due to the gradual growth of the crystals. However, if the sintering time is extended to 4 h, the crystal grains will become coarse, which will significantly affect the performance of the coating materials.

## 4. Conclusions

Homogeneous amorphous glass powder can be obtained after calcined at 1400 °C for 2 h. By using 24.5 wt.% CaO, 10.5 wt.% MgO, 40 wt.% SiO_2_, and 25 wt.% B_2_O_3_ as raw materials, the obtained glass-ceramic has a thermal expansion coefficient of 8.0 × 10^−6^ K^−1^ matching with Al_2_O_3_. The mechanical properties and Vickers hardness of the bulk sample are 2.6 g/cm^3^ and 6.43 GPa, respectively, and its low dielectric constant and loss tangent value meet the performance requirements of the coating on force, heat, and electricity. It can be found that the higher the solid content of the paste, the higher the viscosity and the denser the prepared coating. The 3D-printed coating shows well-bonded interfaces between the Ag layer, the CMS coating, and the Al_2_O_3_ substrate. The diffusion depth was 2.5 μm, and no obvious pores and cracks can be detected. Diopside precipitates can be well formed at 850 °C, and the optimized sintering time was 3 h. Further increasing the sintering temperature and extending the sintering time has negative side effects.

## Figures and Tables

**Table 1:** Main properties of Al_2_O_3_ ceramic substrate.

Permittivity (20 °C, 1 MHz)	Density (kg/cm^3^)	Bending Strength (MPa)	Thermal Conductivity (W/m°C)	Resistivity (Ω·cm)	Thermal Expansion Coefficient (ppm/°C)
9	≥3.7	300	29.3	10^13^	8.0

**Table 2:** Mixture proportion of CaO-MgO-SiO_2_-B_2_O_3_ system.

	CaO (wt.%)	MgO (wt.%)	SiO_2_ (wt.%)	B_2_O_3_ (wt.%)
C1	31.5	13.5	40	15
C2	28	12	40	20
C3	24.5	10.5	40	25
C4	21	9	40	30

**Table 3:** Properties of CMS-based glass-ceramic with various components.

Sample	Bulk Density (g/cm^3^)	Vickers Hardness (GPa)	The Average Coefficient of Thermal Expansion (×10^−6^ K^−1^) (100~500 °C)
C1	2.84	7.36	9.38
C2	2.65	6.73	8.41
C3	2.60	6.63	8.10
C4	2.59	6.52	7.25

## Data Availability

Not applicable.
